# Hypertension, renin-angiotensin-aldosterone-system-blocking agents, and COVID-19

**DOI:** 10.1186/s40885-021-00168-0

**Published:** 2021-06-01

**Authors:** Si-Hyuck Kang, Dong-Hoon Lee, Kyung-Do Han, Jin-Hyung Jung, Sang-Hyun Park, Andrew M. Dai, Henry G. Wei, Chang-Hwan Yoon, Tae-Jin Youn, In-Ho Chae, Cheol-Ho Kim

**Affiliations:** 1 Cardiovascular Center, Department of Internal Medicine, Seoul National University Bundang Hospital, Seongnam, Republic of Korea; 2 Department of Internal Medicine, Seoul National University College of Medicine, Seoul, Republic of Korea; 3 Google, CA Mountain View, USA; 4 Department of Statistics and Actuarial Science, Soongsil University, Seoul, Republic of Korea; 5 Department of Biostatistics, College of Medicine, The Catholic University of Korea, Seoul, Republic of Korea

**Keywords:** Coronavirus infections, Hypertension, Angiotensin-converting enzyme inhibitors, Angiotensin receptor antagonists

## Abstract

**Background:**

There have been concerns regarding the safety of renin-angiotensin-aldosterone-system (RAAS)-blocking agents including angiotensin-converting enzyme inhibitors (ACEI) and angiotensin receptor blockers (ARB) during the coronavirus disease 2019 (COVID-19) pandemic. This study sought to evaluate the impact of hypertension and the use of ACEI/ARB on clinical severity in patients with COVID-19.

**Methods:**

A total of 3,788 patients aged 30 years or older who were confirmed with COVID-19 with real time reverse transcription polymerase chain reaction were identified from a claims-based cohort in Korea. The primary study outcome was severe clinical events, a composite of intensive care unit admission, need for ventilator care, and death.

**Results:**

Patients with hypertension (n = 1,190, 31.4 %) were older and had higher prevalence of comorbidities than those without hypertension. The risk of the primary study outcome was significantly higher in the hypertension group, even after multivariable adjustment (adjusted odds ratio [aOR], 1.67; 95 % confidence interval [CI], 1.04 to 2.69). Among 1,044 patients with hypertensive medical treatment, 782 (74.9 %) were on ACEI or ARB. The ACEI/ARB subgroup had a lower risk of severe clinical outcomes compared to the no ACEI/ARB group, but this did not remain significant after multivariable adjustment (aOR, 0.68; 95 % CI, 0.41 to 1.15).

**Conclusions:**

Patients with hypertension had worse COVID-19 outcomes than those without hypertension, while the use of RAAS-blocking agents was not associated with increased risk of any adverse study outcomes. The use of ACE inhibitors or ARBs did not increase the risk of adverse COVID-19 outcomes, supporting current guidance to continue these medications when indicated.

## Background

The coronavirus disease 2019 (COVID-19) is a global pandemic causing millions of deaths worldwide as of March 2021 [1, 2]. The pathogen of the disease, severe acute respiratory syndrome coronavirus-2, uses a densely glycosylated spike (S) protein to gain entry into host cells [3]. Studies have suggested that the host receptor angiotensin-converting enzyme 2 (ACE2) is the target of the S protein receptor-binding domain, and that the interaction regulates the transmission of the disease [4, 5].

Amid the COVID-19 pandemic, concerns have been raised that the use of renin-angiotensin-aldosterone-system (RAAS)-blocking agents, such as ACE inhibitors (ACEI) and angiotensin receptor blockers (ARB) may increase the susceptibility to or aggravate the severity of COVID-19. There have been studies suggesting blockade of either angiotensin II synthesis or activity increases the gene expression and activity of ACE2 [6, 7]. Observations that patients with hypertension are associated with worse clinical outcomes triggered the concerns that the use of ACEI or ARB may be linked with their poor prognosis.

Major cardiology societies pointed out that such speculations lack sound clinical evidence, and strongly recommended that physicians and patients should continue treatment with their usual antihypertensive therapy [8, 9]. Hypertension is one of the most common diseases, and strong evidence supports the benefit of medical treatment for hypertension [10–12]. ACEI and ARB are among the most widely used antihypertensive medications. In contrary to the initial concerns, multiple studies have proven their safety in terms of COVID-19 susceptibility and severity [13, 14]. This study aimed at estimating the impact of hypertension and the use of ACEI/ARB on severe clinical outcomes among Korean patients confirmed with COVID-19.

## Methods

### Data source and patients

South Korea is one of the countries severely affected by COVID-19: more than 20,000 patients have been diagnosed and approximately 380 died as of September 2020. In response to scientific needs, the government of Korea allowed access to de-identified COVID-19 nationwide patient data based on the Korean National Health Insurance System [15]. The insurance system covers more than 97 % of residents in Korea. The cohort comprised 5,483 patients confirmed by real time reverse transcription polymerase chain reaction (RT-PCR) until April 4, 2020. Hospitalized patients as well as those treated at outpatient clinics were included. The database consisted of claims information during the COVID-19 illness and the previous 5 years. Adults aged 30 years or older (n = 3,788) were chosen in this study because hypertension is rare in younger populations [16]. This study was exempt from review by the Seoul National University Bundang Hospital Institutional Review Board (IRB No: X-2005-611-902). It complied with the requirements of the 2013 Declaration of Helsinki, and the need for informed consent was waived.

### Definitions

A subject was considered to have hypertension if (a) hypertension was diagnosed once or more during a hospitalization, or at two or more outpatient clinic visits; or (b) a history of antihypertensive medication prescription for more than 1 month during the prior 1 year. Subjects with antihypertensive medical treatment were classified into an ACEI/ARB or non-ACEI/ARB group according to the prescription in the year prior: ACEI/ARB use was necessary regardless of combined use of other antihypertension medications to suffice the ACEI/ARB group, while non-ACEI/ARB group definition included any antihypertension drugs other than ACEI or ARB. Previous medical history, such as ischemic heart disease, stroke, congestive heart failure, chronic obstructive pulmonary disease, and cancer, was identified based on International Classification of Diseases-10 codes from the previous 5-year claim data [17]. The primary study outcome was a composite of intensive care unit (ICU) admission, need for ventilator care, and death. Secondary outcomes included hospitalization, oxygen requirement, ICU admission, need for ventilator care, and death related to COVID-19.

### Statistical analysis

Summary statistics are reported as means ± standard deviations for numerical variables and as numbers (percentages) for categorical variables. Continuous variables were compared using the Student t-test or the Mann-Whitney U-test, as appropriate. Categorical variables were compared using the chi-squared test. Logistic regression models were used to calculate odds ratios (ORs) and 95 % confidence intervals (CIs). Multivariable regression models were constructed with adjustment for (a) age (continuous) and sex, and (b) including age, sex, diabetes mellitus, chronic kidney disease, chronic obstructive pulmonary disease, and cardiovascular disease. A two-sided *P* < 0.05 was considered statistically significant. Statistical analyses were performed using SAS ver. 9.2 (SAS Institute, Cary, NC, USA).

## Results

### Hypertension and COVID-19 outcomes

**Fig. 1:**
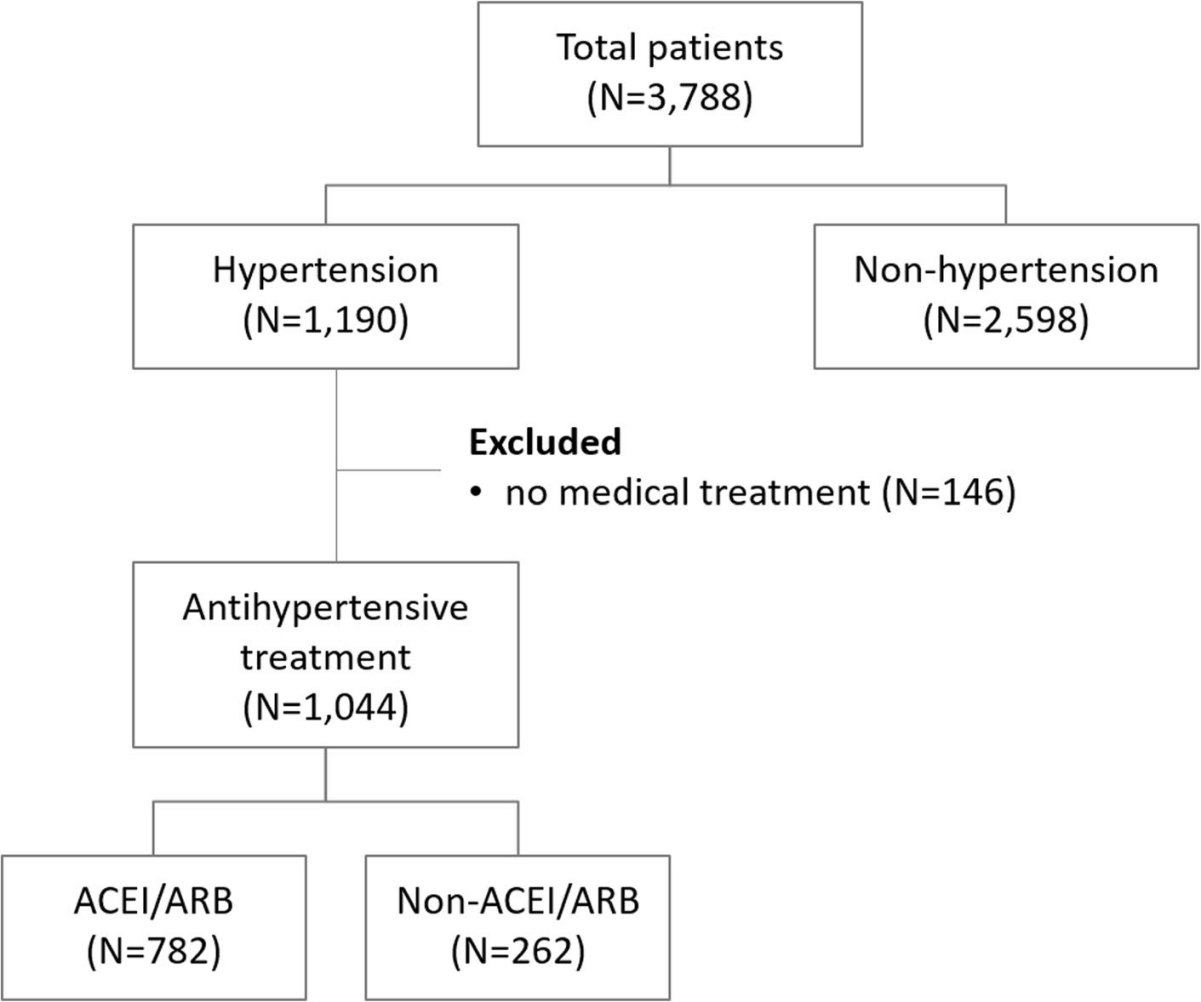
Study flow diagram. ACEI, angiotensin-converting enzyme inhibitors; ARB, angiotensin receptor blockers

Among 3,788 adults aged 30 years or older who were diagnosed with COVID-19, 1,190 (31.4 %) had hypertension (Fig. 1). As shown in Table 1, the two groups showed significantly different age distributions. While the number peaked at 30 to 39 years and decreased linearly with an increasing age in the non-hypertension group, the hypertension group showed a broader peak between 50 and 79 years. The proportions of male sex, diabetes, chronic kidney disease, chronic obstructive pulmonary disease, cardiovascular disease, and cancer were also higher in the hypertension group.

**Table 1:** Baseline characteristics of study population

Characteristic	Total (*n* = 3,788)	Hypertension (*n* = 1,190)	Non-hypertension (*n* = 2,598)	*P*-value
Age (yr)	52.1 ± 15.1	63.9 ± 14.0	46.7 ± 12.3	< 0.001
Age distribution (yr)				< 0.001
30‒39	970 (25.6)	57 (4.8)	913 (35.1)	
40‒49	860 (22.7)	142 (11.9)	718 (27.6)	
50‒59	808 (21.3)	259 (21.8)	549 (21.1)	
60‒69	570 (15.0)	289 (24.3)	281 (10.8)	
70‒79	374 (9.9)	271 (22.8)	103 (4.0)	
80–89	180 (4.8)	151 (12.7)	29 (1.1)	
≥ 90	26 (0.7)	21 (1.8)	5 (0.2)	
Male sex	1,692 (44.7)	605 (50.8)	1,087 (41.8)	< 0.001
Comorbidity				
Diabetes	655 (17.3)	483 (40.6)	172 (6.6)	< 0.001
Dyslipidemia	1,103 (29.1)	716 (60.2)	387 (14.9)	< 0.001
Chronic kidney disease	234 (6.2)	213 (18.0)	21 (0.8)	< 0.001
Chronic obstructive pulmonary disease	387 (10.2)	213 (18.0)	174 (6.7)	< 0.001
Cancer	313 (8.3)	153 (12.9)	160 (6.2)	< 0.001
Cardiovascular disease	513 (13.5)	429 (36.1)	84 (3.2)	< 0.001
Ischemic heart disease	269 (7.1)	226 (19.0)	43 (1.7)	< 0.001
Myocardial infarction	36 (1.0)	34 (2.9)	2 (0.1)	< 0.001
Stroke	185 (4.9)	163 (13.7)	22 (0.8)	< 0.001
Congestive heart failure	223 (5.9)	191 (16.1)	32 (1.2)	< 0.001

Data are presented as mean ± standard deviation or number (%)

**Fig. 2:**
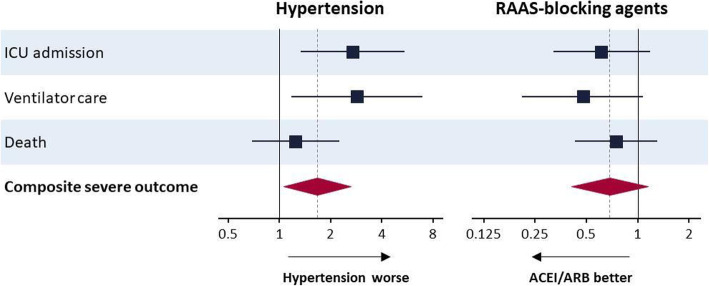
Risk of adverse clinical outcomes associated with hypertension and the use of RAAS-blocking agents. RAAS, renin-angiotensin-aldosterone-system; ACEI, angiotensin-converting enzyme inhibitors; ARB, angiotensin receptor blockers

The hypertension group showed significantly poor outcomes compared to the non-hypertension group (Table 2; Fig. 2). ORs for the primary study outcome, a composite of ICU admission, ventilator care, and death, were 5.18 (95 % CI, 3.51 to 7.65) before adjustment and 1.67 (1.04 to 2.69) after multivariable adjustment. While hospitalization and oxygen requirement also occurred more frequently, they were not significant after adjustment.

**Table 2:** Clinical outcomes according to the presence of hypertension

Clinical outcome	Total (*n* = 3,788)	Hypertension (*n* = 1,190)	Non-hypertension (*n* = 2,598)	Unadjusted		Age, sex-adjusted		Multivariable adjusted	
				OR (95 % CI)	*P*-value	OR (95 % CI)	*P*-value	OR (95 % CI)	*P*-value
Hospitalization	1,481 (39.1)	587 (49.3)	894 (34.4)	1.86 (1.61‒2.13)	< 0.001	0.96 (0.81‒1.14)	0.634	1.15 (0.95‒1.38)	0.146
Oxygen requirement	267 (7.0)	154 (12.9)	113 (4.3)	3.27 (2.54‒4.21)	< 0.001	1.24 (0.92‒1.68)	0.159	1.33 (0.96‒1.83)	0.083
Severe outcome	123 (3.2)	85 (7.1)	38 (1.5)	5.18 (3.51‒7.65)	< 0.001	1.66 (1.06‒2.59)	0.026	1.67 (1.04‒2.69)	0.034
ICU admission	55 (1.5)	38 (3.2)	17 (0.7)	5.01 (2.82‒8.91)	< 0.001	2.59 (1.33‒5.05)	0.005	2.69 (1.33‒5.43)	0.006
Ventilator care	34 (0.9)	25 (2.1)	9 (0.3)	6.17 (2.87‒13.3)	< 0.001	2.50 (1.05‒5.94)	0.038	2.85 (1.18‒6.92)	0.020
Death	83 (2.2)	61 (5.1)	22 (0.8)	6.32 (3.87‒10.3)	< 0.001	1.36 (0.78‒2.36)	0.274	1.24 (0.69‒2.25)	0.473

Data are presented as number (%) unless otherwise specified

*ICU* intensive care unit, *OR* odds ratios, *CI* confidence intervals

### RAAS-blocking agents and COVID-19 outcomes

Regarding antihypertensive medical treatment, 782 out of 1,044 hypertensive adults (74.9 %) were prescribed ACEI or ARB (Fig. 1). The ACEI/ARB group showed a younger age and higher frequencies of diabetes, dyslipidaemia, and chronic kidney disease, while the proportion of cancer was significantly lower than the non-ACEI/ARB group (Table 3; Fig. 2). Meanwhile, the two groups showed disparate patterns in antihypertensive medical treatment.

**Table 3:** Comparison of baseline characteristics of hypertensive adults according to antihypertensive treatment

Characteristic	Antihypertensive treatment (n = 1,044)	ACEI/ARB (n = 782)	Non-ACEI/ARB (n = 262)	*P*-value
Age (yr)	63.8 ± 14.1	63.0 ± 14.2	66.2 ± 13.6	0.002
Male sex	525 (50.3)	401 (51.3)	124 (47.3)	0.268
Comorbidity				
Diabetes	410 (39.3)	333 (42.6)	77 (29.4)	0.002
Dyslipidemia	620 (59.4)	481 (61.5)	139 (53.1)	0.016
Chronic kidney disease	177 (17.0)	144 (18.4)	33 (12.6)	0.030
Chronic obstructive pulmonary disease	181 (17.3)	134 (17.1)	47 (17.9)	0.766
Cancer	119 (11.4)	80 (10.2)	39 (14.9)	0.040
Cardiovascular disease	358 (34.3)	261 (33.4)	97 (37.0)	0.282
Ischemic heart disease	185 (17.7)	137 (17.5)	48 (18.3)	0.769
Myocardial infarction	27 (2.6)	23 (2.9)	4 (1.5)	0.212
Stroke	137 (13.1)	97 (12.4)	40 (15.3)	0.235
Congestive heart failure	163 (15.6)	113 (14.5)	50 (19.1)	0.074
Antihypertensive treatment				
ACEI	51 (4.9)	51 (6.5)	0	< 0.001
ARB	744 (71.3)	744 (95.1)	0	< 0.001
β-blockers	183 (17.5)	122 (15.6)	61 (23.3)	0.005
Calcium channel blockers	593 (56.8)	422 (54.0)	171 (65.3)	0.001
Diuretics	223 (21.4)	187 (23.9)	36 (13.7)	0.001
Others	100 (9.6)	48 (6.1)	52 (19.8)	< 0.001

Data are presented as mean ± standard deviation or number (%)

*ACEI* angiotensin-converting enzyme inhibitors, *ARB* angiotensin receptor blockers

Table 4 shows unadjusted and multivariable-adjusted risk estimates for the study outcomes. The primary endpoint occurred in a lower rate in the ACEI/ARB group before adjustment, which lost significance after adjustment (OR, 0.68; 95 % CI, 0.41 to 1.15). While ICU admission and ventilator care tended to be lower in the ACEI/ARB group with marginal significance, no other study endpoints differed significantly.

**Table 4:** Clinical outcomes according to the use of RAAS blockers

Clinical outcome	Antihypertensive treatment (*n* = 1,044)	ACEI/ARB (*n* = 782)	Non-ACEI/ARB (*n* = 262)	Unadjusted		Age, sex-adjusted		Multivariable adjusted	
				OR (95 % CI)	*P*-value	OR (95 % CI)	*P*-value	OR (95 % CI)	*P*-value
Hospitalization	540 (51.7)	389 (49.7)	151 (57.6)	1.05 (0.83–1.33)	0.691	1.13 (0.89–1.45)	0.320	1.16 (0.90–1.49)	0.263
Oxygen requirement	142 (13.6)	97 (12.4)	45 (17.2)	0.87 (0.61–1.24)	0.445	0.96 (0.67–1.37)	0.802	0.96 (0.67–1.38)	0.813
Severe outcome	76 (7.3)	49 (6.3)	27 (10.3)	0.58 (0.36–0.95)	0.031	0.66 (0.40–1.09)	0.104	0.68 (0.41–1.15)	0.150
ICU admission	33 (3.2)	20 (2.6)	13 (5.0)	0.57 (0.30–1.09)	0.084	0.60 (0.31–1.16)	0.127	0.61 (0.32–1.18)	0.139
Ventilator care	21 (2.0)	12 (1.5)	9 (3.4)	0.47 (0.21–1.05)	0.059	0.51 (0.23–1.14)	0.101	0.48 (0.21–1.08)	0.076
Death	54 (5.2)	35 (4.5)	19 (7.3)	0.69 (0.41–1.16)	0.161	0.78 (0.45–1.33)	0.358	0.75 (0.43–1.30)	0.302

Data are presented as number (%) unless otherwise specified

*RAAS* renin-angiotensin-aldosterone-system, *ACEI* angiotensin-converting enzyme inhibitors, *ARB* angiotensin receptor blockers, *ICU* intensive care unit, *OR* odds ratios, *CI* confidence intervals

## Discussion

The present study showed patients with hypertension more frequently experienced adverse outcomes than those without hypertension when they have COVID-19 illness. The excess was greater with severe events such as ICU admission, ventilator care, and death, whereas it was less prominent with less severe events such as hospitalization and oxygen requirement. Another important finding of this study is that the use of RAAS-blocking agents such as ACEI and ARB was not associated with increased risk of poor clinical prognosis associated with COVID-19 illness.

The present study showed that use of RAAS-blocking agents does not adversely affect clinical outcomes during COVID-19 illness. Several recent studies consistently showed reassuring results regarding the safety of ACEI/ARB during the pandemic. Two studies from China also analyzed hospitalized patients and found no excess in mortality associated with the use of ACEI/ARB [18, 19]. Several studies even suggested lower risk of mortality associated with ACEI/ARB use [19, 20].

The current evidence of ACE2 upregulation after chronic exposure to ACEI and ARB is mostly focused on cardiovascular tissue [6, 7]. There is still no evidence that they increase lung-specific ACE2 expression [13]. In addition, experimental animal models have shown mixed findings suggesting the complexity underlying RAAS responses to pathway modulators [13]. The present study provides clinical evidence supporting that patients who are taking ACEI or ARB should not discontinue their current treatment during the COVID-19 pandemic.

Previous studies have shown that hypertension is the most frequent comorbidity among patients with COVID-19 and that it is also frequent in those with adverse clinical outcomes [2, 21]. This study also showed hypertension poses a risk factor for poor prognosis in patients with COVID-19. They were also significantly older and had higher prevalence of comorbidities. Although adjustment for such factors partially attenuated the risks, they remained significantly greater.

This study similarly showed hypertension was the most frequent comorbidity in this study population. However, the proportion (31.4 %) was numerically similar to the prevalence in the general Korean population (30.5 % in adults aged 30 years or older) [16, 22]. Regarding to the role of RAAS-blocking agents, Mancia et al. [14] recently performed a well-designed case-control study to demonstrate that ACEI or ARB did not affect the risk of COVID-19. Subsequent studies also confirmed the safety of ACEI and ARB in terms of likelihood of COVID-19 infection [23, 24].

One of the strengths of the present study is the broad spectrum of study population including outpatients as well as in-hospital patients. Vigorous contact-tracing and extensive testing were performed in Korea [25]. The low rate of mortality (2.2 %) in this study compared to other studies with RAAS-blocking agents (5.8 %, 8.8 %, and 11.0 %) partly reflects the situation [18, 19]. We believe it ensures low risk of selection bias in this study. However, this study also has limitations. Firstly, because the cohort consisted of a claims database, study variables were defined using operational definitions. However, the use of claims data also ensures the data integrity of COVID-19-related adverse outcomes and history of antihypertensive medication usage. Second, while information on ethnicity was absent, it is likely that most of the patients were ethnic Korean. The narrow ethnic representativeness is another limitation. Third, ARB outnumbered ACEI in the study cohort reflecting the practice pattern in East Asia. High prevalence of adverse events such as dry cough has been reported with the use of ACEI in the region [26]. Lastly, claims data usually have lag time until registration. It is possible that recent information on medical usage may be incomplete yet.

## Conclusions

This study showed hypertension is associated with poor clinical outcomes in patients with COVID-19. However, the use of RAAS-blocking agents, including ACEI and ARB, was not associated with increased risk of any adverse study outcomes. The present study supports the current guidance to continue ACEI or ARB during the COVID-19 pandemic.

## Data Availability

Not applicable.

## Abbreviations

ACE: Angiotensin-converting enzyme
ACEI: Angiotensin-converting enzyme inhibitors
ARB: Angiotensin receptor blockers
CI: Confidence interval
COVID-19: Coronavirus disease 2019
ICU: Intensive care unit
OR: Odds ratio
RAAS: Renin-angiotensin-aldosterone-system
S: Spike
